# Characterizations and Electrical Modelling of Sensory Samples Formed from Synthesized Vanadium (V) Oxide and Copper Oxide Graphene Quantum Tunneling Composites (GQTC) Applied in Electrotribology

**DOI:** 10.3390/s16010058

**Published:** 2016-01-05

**Authors:** Tadeusz Habdank-Wojewódzki, Josef Habdank, Przemyslaw Cwik, Slawomir Zimowski

**Affiliations:** 1 AGH University of Science and Technology, Department of Electronics, Mickiewicza 30, 30-059 Krakow, Poland; habdankw@agh.edu.pl; 2 Graphenalloy, Ordrupvej 69, 3th, 2920 Charlottenlund, Denmark; 3 Delphi Automotive Systems, Powstancow Wielkopolskich 13 D-E, 30-962 Krakow, Poland; przemyslaw.cwik@gmail.com; 4 AGH University of Science and Technology, Faculty of Mechanical Engineering and Robotics, Mickiewicza 30, 30-059 Krakow, Poland; zimowski@imir.agh.edu.pl

**Keywords:** semiconductive metal oxide composites, thermopiezoresistors, quantum tunneling composites (QTC), graphene, graphene quantum tunneling composites (GQTC), electrotribology, MEMS

## Abstract

CuO and V_2_O_5_ graphene quantum tunneling composites (GQTC) presented in this article were produced and their sensory properties were analyzed. The composites were synthesised using two stage high-power milling process, which resulted in materials that have good temeprature and pressure sensory properties. Described production process defines internal structure of materials such that when used as sensor in the desired range, it exhibits a strong percolation effect. The experiment, with controlled changing physical conditions during electrotribological measurement, enabled analyzing of the composites’ conductivity as a function of the sensory properties: applied temperature, pressure, tangential force and wear. The sensory characteristic was successfully modelled by invertible generalized equations, and used to create sensor capable of estimating temperature or pressure in the real time. The developed materials have the potential to be applied in the areas where miniaturization is essential, due to the materials exhibiting good sensory properties in mini and micro scale.

## 1. Introduction

Quantum tunneling composites (QTC) exhibiting very strong piezoresistive [1,2,3,4,5,6] as well as thermoresistive [7,8,9,10,11] sensory properties undergone extensive research in the last five years. The initial discovery and coining of the term was done [12,13,14,15,16] and brought the interest of a wide range of researchers. Initial focus was targeted towards tactile sensors for the robots [2,17,18,19,20,21] which require very high sensitivity, but, later, the interest moved towards thermistors.

The temperature coefficient of resistivity (TCR) of QTC was shown to be orders of magnitude better than the platinum [22], which is the industry baseline, and, for some cases, is as high as 10%–40%/K [9,23]. However, those materials with giant temperature coefficients exhibit extremely non-linear and non-consistent behavior and are hard to accurately model and reproduce.

Composites presented in this article differ from the above as they use the graphene nanoplatelets [24,25] among other fillers as a basis for production. Graphene quantum tunneling composites (GQTC) are synthesized using two stage high-power milling process [26,27,28], and are characterized by the critical filler density of the percolation curve [29]. GQTC can be characterized by smooth conductance space which is more accurate than QTC [30] and can be successfully modelled by invertible equations, which enables them to be reliably used as sensors. The materials’ behavior is analyzed both when used as a static sensor which is not disturbed by any external processes, as well as when operating as an element of a friction node which disturbs the measurement by stochastic or chaotic processes occurring on the contact. The purpose of the research is to validate the materials for the application as piezoresistive and thermoresistive sensors operating both in perturbed and non-perturbed environments. In order to analyze these materials and their behavior, a special measuring station (called electrotribotester) was designed which allows measuring a wide range of parameters of the materials.

Although both piezo and thermoresistive properties are known and widely described in many papers on what contributes to achieve good sensor material, only few works analyze and discuss piezoresistivness and thermoresistiveness at the same time. Comprehensive published knowledge of both abovementioned features discovers a full spectrum of sensory applications that are possible. Moreover, many investigators deeply analyze tribological performance of graphene nanocomposites [31,32] and explore friction and wear influence on electrical conductivity [33,34]. Nonetheless, most of the published experiments’ shortcomings are a lack of analysis of prototype conditions and phenomenons such as an influence of dynamic electrical contact.

This study fills the gap between aforementioned extensive research and discuss electrical characteristics of exceptional piezo and thermoresistive material undergoing shear forces due to friction. This work can be considered unique, as no research specifically targeting practical applications could be found.

## 2. Preparation and Internal Structure of the GQTC

The sensory GQTC consists of three main components: insulating matrix such as resin, one or more semiconductive sensory fillers and graphene. The process consists of high power dry-milling of the filler powder (CuO from Sigma Aldrich, V_2_O_5_ from Avantor Performance Materials) milled for 5 h, which reduces the grain size to ~200 nm in diameter. Then, resulting powder is high power milled for 15 min with graphene nanoplatelets (from Graphene Supermarket). The resulting powder is mixed with polyester resin NH91LV (from Milar Sp. z o.o., Grodzisk Mazowiecki, Poland) and homogenized by using a triple roller mill. The resulting paste is formed into a sensor in a two-part polytetrafluoroethylene mold. The element is thermally set in a temperature of 220 °C for 3 h.

**Figure 1:**
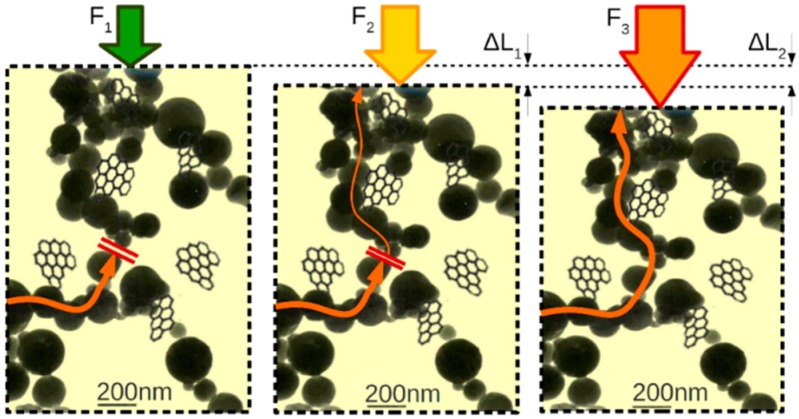
Cartoon drawing depicting three states of conductive chain, changing due to elastic deformation of the structure: (**left**) No conductance; (**middle**) Tunneling barrier, partial conductance; (**right**) Contact and full conductance. Similar process of barrier thinning occurs with the increase of the temperature. Graphene nanoplatelets not to scale.

The CuO and V_2_O_5_ fillers were chosen as they are well known for their thermosresistive and piezoresistive properties [35,36,37,38]. The filler density is experimentally chosen to be close to, but below, the percolation threshold of two statistical contacts per grain. In such materials, even a small perturbation of the crystalline structure of the material significantly increases the probability of tunneling across the barrier of the insulating matrix. This process amplifies the sensory properties of the fillers, thanks to the change-induced statistical formation of conductive chains. Each barrier, within a potential conductive chain, can be in one of three states: no conductance, tunneling barrier (partial conductance), and contact (full conductance) (Figure 1).

## 3. Experimental Design

The measuring station, called an electrotribotester, is a device which allows for measuring the changes in the conductivity of the GQTC sample when subjected to a range of temperatures, external pressures, tangential forces and wear. Such a device allows analysis of the material as a function of the standard parameters of a friction node (pressure, velocity, temperature: PvT), extending it with a range of related signals. The device can operate in two main modes:

- static: performing measurements of conductivity, admittance, I-V curve as a function of temperature and pressure.
- dynamic: subjecting the sample to the wear process, performing measurements of conductivity, admittance, I-V curve as well as temperature (shaft’s surface temperature measured by pyrometer and two thermocouples measuring the sample), pressure, vibrations (two 2D accelerometers), tangential force (the tensometer), rotational speed of the shaft as well as thickness of the sample as a function of time.

The goal of performing both static and dynamic measurements is to research the difference of material’s behavior when subjected to forces which alter the internal structure (internal stresses of the material) and contact conductance, and to test the applicability of the material as a sensor.

**Figure 2:**
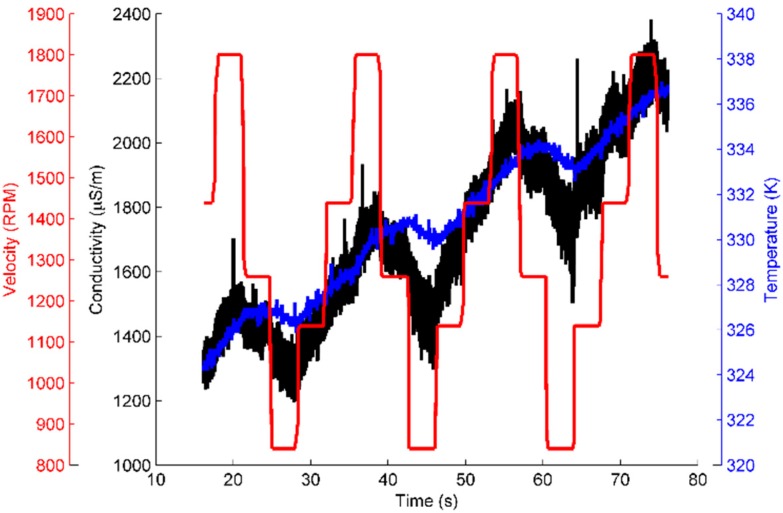
An example of a dynamic measurement done on the electrotribotester, with measured conductivity, temperature and rotational velocity of the shaft causing the wear.

Figure 2 shows a strong correlation between conductivity, temperature and rotational velocity (which changes the tangential force caused by the friction): as the temperature rises, the conductivity rises. The relationship between the conductivity and rotational velocity can be most clearly seen for times from 65 to 75 s, where the conductance clearly changes in a step-like fashion, as the rotational velocity is. It should be noted that the conductivity changes lead changes in the temperature, which is due to temperature having a slower response time caused by the heat capacity of the material.

## 4. Analysis and Modelling of the Conductivity-PvT Space

**Figure 3:**
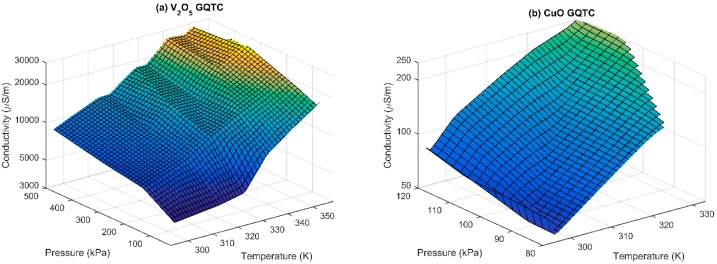
Observed (measured) conductivity σ(P,T) of (**a**) V_2_O_5_; and (**b**) CuO GQTC for static measurements.

Due to the exponential trend of the process of forming of the conductivity chains, the data is analyzed in the logarithmic conductivity space. This transformation linearizes the space (Figure 3), which makes the modelling of the data significantly simpler. Observed conductivity (in log space) of CuO and V_2_O_5_ GQTC is semi-linearly increasing both for the temperature and pressure.

**Figure 4:**
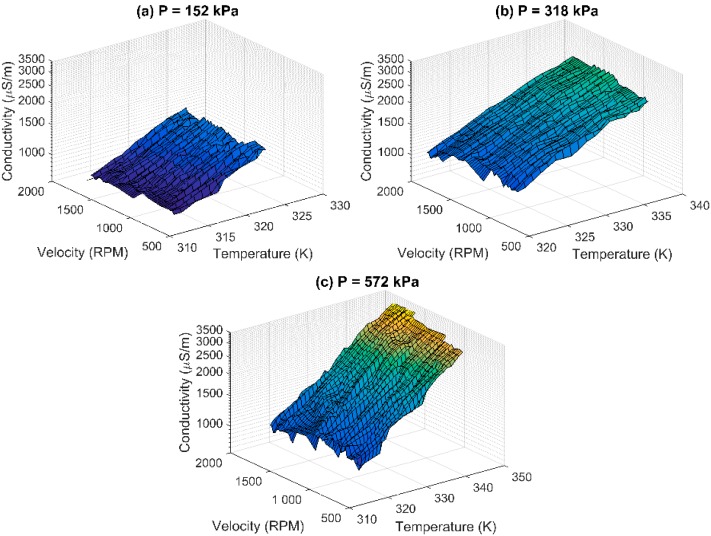
Observed (measured) conductivity σ(*P,v,T*) of CuO GQTC for dynamic measurements for (**a**) pressure P = 152 kPa; (**b**) pressure P = 318 kPa; and (**c**) pressure P = 572 kPa.

Due to the CuO GQTC having less fluctuations and acting more linearly in the σ(*P,T*) space, it was chosen for dynamic analysis, when being subjected to wear and tear (Figure 4). Changes in the rotational velocity cause changes in the friction force, which, in turn, causes elastic changes in the material thus impacting the thickness of the barriers, which affects the conductivity. Similarly, the material exhibits semi-linear behavior.

**Figure 5:**
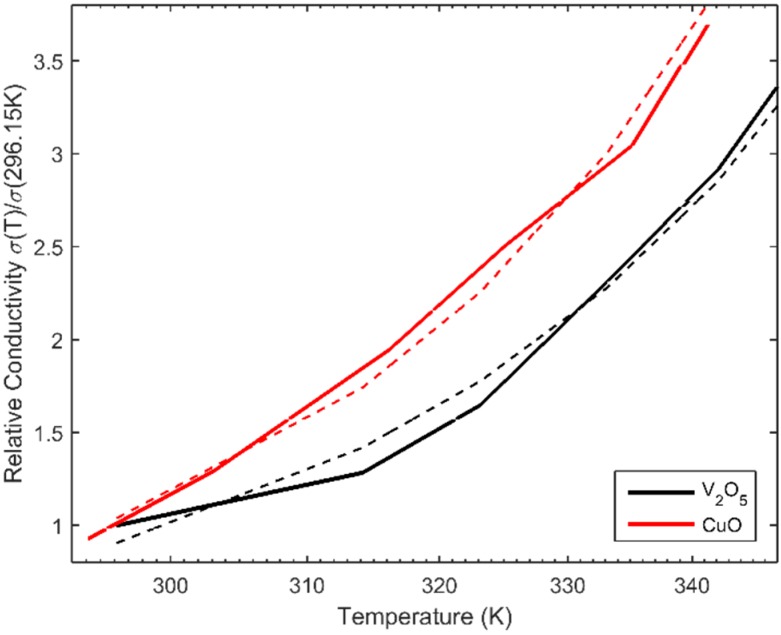
Comparison of the relative conductivity σ(T) for P = 124 kPa for both CuO and V_2_O_5_ GQTC.

From the sensory perspective, both materials exhibit very good sensitivity, with the relative conductivity increasing ~3.5 fold over the change in temperature of ~50 K (Figure 5).

The standart measure of sensitivity of a termistor is a temperature coefficient of resistance (TCR), which is expressed by the following formula:

(1) $$TCR=\frac{1}{R_{0}}\frac{dR}{dT}\cdot 100\%\;\text{in}\;\%/K$$

For TCR, the values computed for *P* = 124 kPa are shown in Table 1.

**Table 1:** Temperature coefficients of resistance (TCRs) of the composites.

Composite	Dynamic/Static	TCR [%/K]
CuO	v = 0	1.7
CuO	v ≠ 0	1.5
V_2_O_5_	v = 0	1.3

The nature of QTC materials allows obtaining very high TCR such as in [2] where TCR ranges from 10%/K to 40%/K. However, in such materials, the resistivity is much less predictable, thus making them hard to use in sensors estimating precise temperature or pressure. Such giant resistivity changes are good for sensors with on/off type of characteristics. Our materials exhibit both very high TCR and very predictable resistance thus making them excellent for sensory application.

### 4.1. Implications of the Material’s Structure on the Modelling

Formation of the conductive chains, described in Section 2 and depicted in Figure 1, is an inherently sigmoidal process and is well researched and documented [2,39]. The resistivity of the composite is proportional to the area of a combined cross section of all the conductive chains.

(2) $$\rho \approx R\frac{\sum_{n=0}^{N}A_{n}}{l}$$

where $A_{n}$ is the cross section area of each of the conductive chains. As the conductive chains are formed with the probability, which is a sigmoid function of the filler density $f_{d}$, and the sum of area of the chains is the expected value of their probabilities, the resistivity will as well exhibit sigmoidal trends.

(3) $$\rho \approx R\frac{N\mu \left(p_{A}\right)}{l},\;p_{A}=sig\left(f_{d}\right)$$

As the filler density $f_{d}$ can be expressed as a statistical average distance between the grains, which is a function of parameters which modify the thickness of the barrier (in this case pressure, velocity and temperature), the final resistivity will be a sigmoidal function.

(4) ρ~sig(P, v, T)

In cases when the process of interest is modelled in a part of the domain, the sigmoid function can be approximated by an exponential function. This approximation is used in many currently used, well documented models of sensing materials such the Steinhart–Hart model for the thermistor [40]:

(5) $$A+Bln\rho +Cln^{3}\rho =\;T^{-1}$$

as well as models proposed for QTC materials such as the QTC resistivity [9]:

(6) $$\text{ln}\left(\rho \cdot A\right)=\;B\omega \left(p\right)\cdot {\left(T+C\right)}^{-1}$$

where, ρ is the resistivity, ω(p) is the average distance between the conductive grains and T is the temperature of the composite.

### 4.2. Empirical Modelling of the Conductivity-PvT Space

The observed phenomena were approximated using an empirical model based on the knowledge described in the previous section. Empirical modelling partially based on physical knowledge and partially on numerical approximation is very useful for practical applications, as they can deliver very accurate estimations of the data as well as enabling inversion of the equations. The proposed general model for conductivity of GQTC is:

(7) $$\overset{M}{\underset{m=0}{\sum }}\overset{Q}{\underset{q=0}{\sum }}b_{q}p_{m}^{sq}=\overset{N}{\underset{n=0}{\sum }}a_{n}ln^{n}\left(\sigma \right)$$

where:

- σ is conductivity, but can be conductance, resistance or resistivity
- *p_m_* is any parameter that σ depends on such as *P, v, T*
- *M* is the dimensionality of the domain of the model (in case of *PvT* model, M = 3)
- *Q* is the order of the Taylor series of the domain parameters
- *N* is the order of the logarithmic series of σ
- *a* and *b* are estimated parameters
- *s* is the sign on the domain parameter, 1 for linear space, −1 for inverse space

This model is based on observation that the nonlinearity of the conductivity space σ can be expressed as a logarithmic series, and all the measured parameters p are approximated by a Taylor series. It should be noted that both models described by Equations (5) and (6) can be rewritten to be special cases or this generalized mode. In order to approximate the material’s conductance space σ(*P,v,T*) of CuO and V_2_O_5_ GQTC sets *M* = 3, *N* = 1 and *s* = 1, thus takes a form:

(8) $$\overset{Q}{\underset{q=0}{\sum }}b_{q}P^{q}+\overset{R}{\underset{r=0}{\sum }}c_{r}v^{r}+\overset{S}{\underset{s=0}{\sum }}d_{s}T^{s}=\overset{N}{\underset{n=0}{\sum }}a_{n}ln^{n}\left(\sigma \right)$$

With optimal values of *Q*, *R* and *S* found by the penalized Stochastic Gradient Descent (SGD) estimator used to fit the model.

### 4.3. Modelling of the Static Data (v = 0)

**Figure 6:**
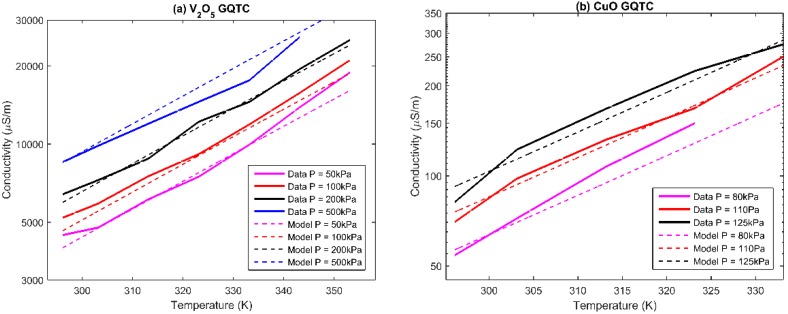
Observed and modelled conductivity of (**a**) the V_2_O_5_ composite; and (**b**) the CuO composite for static (zero velocity) measurements, modelled using σ(*P*,*T*) model.

As seen on the Figure 3, the logarithm of the conductivity space is semi-linear, thus the Taylor series approximation can remain very short. For the V_2_O_5_ GQTC, the penalized SGD estimated the order of *Q* = 2 and *S* = 1, giving Equation (4) for σ(*P,T*).

(9) $$aP+bP^{2}+cT+d=ln\left(\sigma \right)$$

which gives approximation of 91.8% fit (calculated from obtained 8.2% Mean Average Percentage Error, MAPE), shown on Figure 6a. For the CuO GQTC, the penalized SGD estimated the order of *Q* = 1 and *S* = 1, giving Equation (5) for σ(*P,T*).

(10) aP+bT+c=ln(σ)

which gives approximation of 93.7% fit (Figure 6b). It should be noted that the left hand side of the model is a plane, which means the CuO composite behaves close to the exponential.

### 4.4. Modelling of the Dynamic Data (at v ≠ 0)

**Figure 7:**
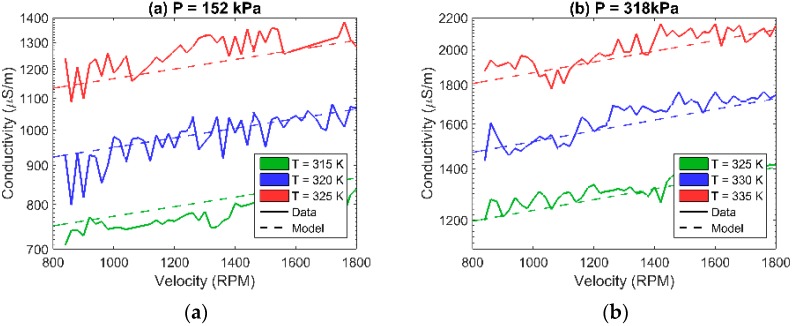

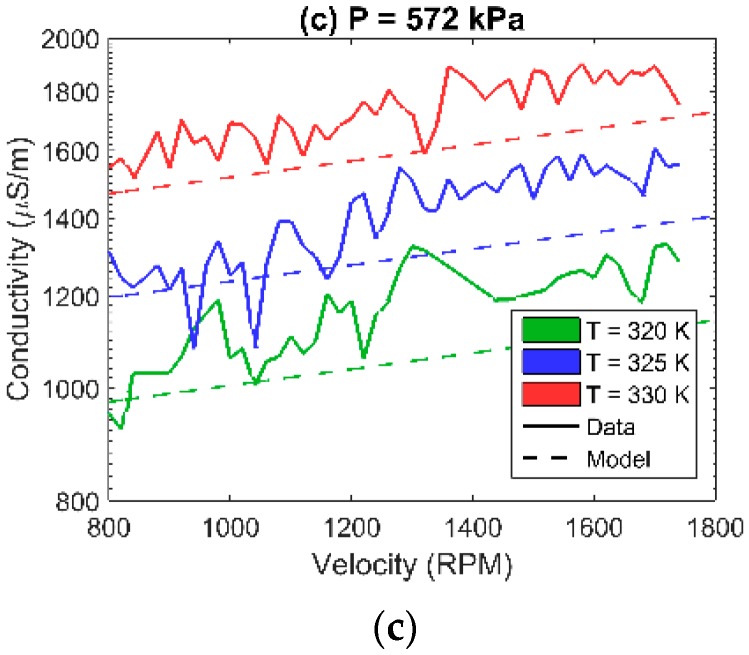
Observed and modelled conductivity of CuO GQTC for dynamic (non-zero velocity) measurements using σ(*P,v,T*) model for (**a**) pressure P = 152 kPa; (**b**) pressure P = 318kPa; and (**c**) pressure P = 572 kPa.

For the dynamic data, the CuO GQTC model extends the domain with the velocity as a parameter. The penalized SGD estimated the order of *Q* = 1, *R* = 1 and *S* = 1 or *Q* = 1, *R* =1 and *S* = 2 as equivalently good. However, taking into account the simplicity of the model, the shorter model is chosen. The model σ(*P,v,T*) takes a form of:

(11) aP+bv+cT+d=ln(σ)

which gives a 95.4% fit. However, in the physical world, pressure and velocity are convolved, as both change the internal structure of the material. When taking this into consideration, the final model σ(*P,v,T*) takes a form of:

(12) aP+bv+cPv+dT+e=ln(σ)

which gives a 96.2% fit (Figure 7).

### 4.5. Real Time Estimation

**Figure 8:**
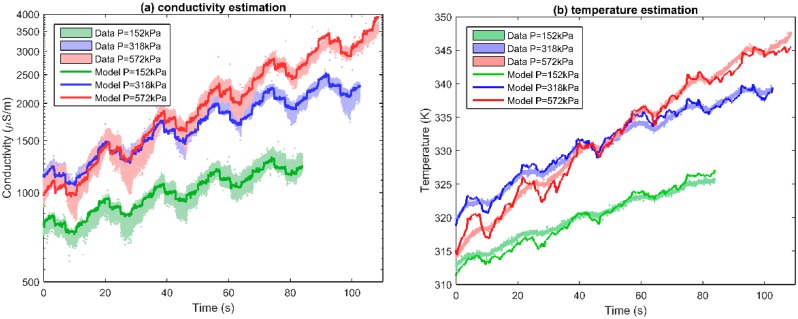
Model estimating (**a**) conductivity σ(*P,v,T*) for CuO GQTC; and (**b**) temperature *T*(*P*,v,σ) for CuO GQTC for the materials subjected wear in time domain.

Since the model has a relatively small error, it can be used to estimate the value of conductivity from the parameters (Figure 8a). As the TCR of the CuO GQTC is high (1.5%/K), and the model can be analytically inverted, the material can be used as a temperature sensor with very high sensitivity and very fast response time (Figure 8b). The model *T*(*P*,*v*,σ) takes a form:

(13) T=aln(σ)+bP+cv+dPv+e

which gives a 98.5% fit.

It should be noted that results presented (Figure 8b) are for the sensor which is subjected to the wear process while doing the measurement. The measured data, due to friction-induced vibrations, stick-slip and other wear related phenomena, has strong components distorting the signal. However, despite these difficult conditions, the temperature estimate is relatively close to the observed value, suggesting that the material can be successfully applied as a sensor in dynamic conditions.

## 5. Conclusions

Composites presented in this article have very good potential to be used in the real-time temperature and pressure sensors, as they have both good sensory properties as well as have easy to model conductivity space. Additionally, the materials have been tested and modelled in an environment with strong distorting signals such as vibrations, friction force and changing contact resistance. Thanks to the proposed model being invertible in respect to all the domain parameters, the same material can be used both as temperature and pressure sensor.

As the sensors produced from the material could operate when undergoing friction and stress, as well as it is possible to make the materials such that they can exhibit very good structural properties (such as low wear and low friction coefficient), it is envisioned that one could produce structural elements from these materials (e.g., bushing, cantilevers, *etc.*). Such elements could be used to self-diagnose its properties such as internal stress, temperature and others. This can be mostly applicable for MEMS, where having external sensors often is impossible due to the scale of the device, and having self-sensing elements could be the only practical way to obtain information about the state of the device itself.
